# Hybrid prevalence estimation: Method to improve intervention coverage estimations

**DOI:** 10.1073/pnas.1810287115

**Published:** 2018-12-05

**Authors:** Caroline Jeffery, Marcello Pagano, Janet Hemingway, Joseph J. Valadez

**Affiliations:** ^a^Department of International Public Health, Liverpool School of Tropical Medicine, L3 5QA Liverpool, United Kingdom;; ^b^Department of Biostatistics, Harvard T. H. Chan School of Public Health, Boston, MA 02115

**Keywords:** HMIS, HIS, health surveys, LQAS, vaccination

## Abstract

Delivering excellent health services requires accurate health information systems (HIS) data. Poor-quality data can lead to poor judgments and outcomes. Unlike probability surveys, which are representative of the population and carry accuracy estimates, HIS do not, but in many countries the HIS is the primary source of data used for administrative estimates. However, HIS are not structured to detect gaps in service coverage and leave communities exposed to unnecessary health risks. Here we propose a method to improve informatics by combining HIS and probability survey data to construct a hybrid estimator. This technique provides a more accurate estimator than either data source alone and facilitates informed decision-making. We use data from vitamin A and polio vaccination campaigns in children from Madagascar and Benin to demonstrate the effect. The hybrid estimator is a weighted average of two measurements and produces SEs and 95% confidence intervals (CIs) for the hybrid and HIS estimators. The estimates of coverage proportions using the combined data and the survey estimates differ by no more than 3%, while decreasing the SE by 1–6%; the administrative estimates from the HIS and combined data estimates are very different, with 3–25 times larger CI, questioning the value of administrative estimates. Estimators of unknown accuracy may lead to poorly formulated policies and wasted resources. The hybrid estimator technique can be applied to disease prevention services for which population coverages are measured. This methodology creates more accurate estimators, alongside measured HIS errors, to improve tracking the public’s health.

## Significance

Over the last 2 decades, low- and middle-income countries have moved from fragmented paper-based systems to electronic health information systems (HIS). Although these are a major advance, they are not well suited to drive operational policy decisions and detect gaps in service coverage. To avoid overreliance on administrative estimates from HIS, probability surveys of population coverage are increasingly used operationally. By combining the probability survey and HIS data, a hybrid prevalence estimate can be generated that can be used to validate and/or moderate HIS administrative estimates of coverage.

Information about a population’s health plays an essential role in planning and managing health systems. Demand by ministries of health and global donors for timely and reliable data continues to increase (1). The 60th World Health Assembly underscored the importance of acquiring robust information to strengthen health systems and policies [resolution WHA60.27 (2)]. For decades, health information records in low- and middle-income countries were fragmented and paper-based, but now they are rapidly moving to electronic systems. The dominant electronic system, supported by international donor communities in 60 countries on four continents, is the District Health Information System-2 (DHIS2). Although DHIS2 should lead to more accurate and rapid use of information for decision-making, it is essential to measure and report its accuracy. Inaccurate information can lead to poor program decisions, unidentified gaps in health service coverage, and the implementation of poorly formulated priorities. The World Health Organization recommends using multiple data sources simultaneously to strengthen decision-making and avoid overreliance on inaccurate administrative statistics. Probability surveys and health information systems are the two most often used data sources.

An important use of health data is to measure population coverage with specific interventions. Typically, coverage is measured by dividing a numerator, which is relatively easy to measure, by a denominator, which is harder to measure (3). Most studies focus on improvements in obtaining numerators. Equally important are accurate and precise denominators and the ability to incorporate a statistically valid method to measure the error of estimates. Without this information, managers are unable to evaluate the risk associated with using the information presented.

Several studies have compared different sources of data to assess the same or similar indicators or used differing information sources in a complementary manner, but they do not combine them in a principled way to routinely produce more robust estimates for an intervention. Although these studies demonstrate differences between health information system (HIS) and survey results, none has measured the error in the HIS, a limitation that many note (1, 4–7). The SE (8) is an estimate of deviation of the sample mean from the actual mean of a population. The SE is used in calculating a 95% confidence interval (CI), which measures the accuracy of the estimate. If survey and HIS data are combined, they produce a single, more accurate coverage estimator (9). Here we propose a method that provides a more accurate hybrid estimator, with a narrower CI than either data source alone.

We carried out household surveys in Benin and Madagascar during the period that administrative data were collected to assess two Child Health Day (CHD) intervention coverages. Using a probability sample and the countries’ HIS data, we produced a hybrid estimator, with its own measure of accuracy, as well as a measure of the accuracy of the administrative HIS data. Our results show that the current HIS-dependent administrative method overestimates CHD coverage and does not detect districts falling short of reaching coverage targets.

## Methods

To demonstrate the theory and practicality of combining HIS and probability survey data to produce a more accurate indicator of health status of a population, we used polio vaccinations and vitamin A supplementation (VAS) data collected through CHD campaigns in Benin and Madagascar in 2015. The method combines (*i*) administrative records of regional managers responsible for managing the CHD (the accuracies of this HIS are unknown) with (*ii*) the results of probability surveys undertaken in the same regions at the same time, whose accuracies are known (10).

### Administrative Data.

Census-quality CHD administrative data were recorded during door-to-door visits targeted at all households. These data, collected differently from facility HIS data, were supplied by the Benin Ministry of Health (MOH) and Madagascar Ministry of Public Health (MOPH) via United Nations International Children's Emergency Fund (UNICEF) country offices.

In Benin, the MOH organizes the administration of VAS and polio vaccination to every eligible child (6–59 mo) via a National Immunization Day (NID) (11). Every 6 mo the VAS and polio vaccination data are tallied nationwide for each of the 85 communes. UNICEF calculated commune-level coverage for both services using this numerator information (Table 1). The denominator of each targeted age group was not known. UNICEF estimated the denominator from the number of children vaccinated during the previous campaign. The distribution of their resulting ratio estimator is what we investigate and report.

**Table 1.** Definition of LQAS and administrative data indicator used in Benin to assess Child Health Days

LQAS indicator	Administrative data indicator
Proportion of children 6–11 mo of age in the probability sample who received a vitamin A supplement during the last campaign (maternal recall, after being shown a vitamin A capsule)	Numerator: number of children 6–11 mo who received a vitamin A supplement during the most recent campaign
	Denominator: number of children 6–11 mo who received a vitamin A supplement during the previous campaign
Proportion of children 12–59 mo of age in the probability sample who received a vitamin A supplement during the last campaign (maternal recall, after being shown a vitamin A capsule)	Numerator: number of children 12–59 mo who received a vitamin A supplement during the most recent campaign
	Denominator: number of children 12–59 mo who received a vitamin A supplement during the previous campaign
Proportion of children 6–11 mo who received polio vaccine during the last campaign (maternal recall)	Numerator: number of children 6–11 mo who received polio vaccine during the most recent campaign
	Denominator: number of children 6–11 mo who received a vitamin A supplement during the previous campaign
Proportion of children 12–59 mo who received polio vaccine during the last campaign (maternal recall)	Numerator: number of children 12–59 mo who received polio vaccine during the last campaign
	Denominator: number of children 12–59 mo who received a vitamin A supplement during the previous campaign

In Madagascar, there are independent annual CHD campaigns for VAS and polio vaccinations (12) (Table 2). The denominator of each targeted age group differs for the two campaigns; the VAS denominator is estimated from the 1993 census data and extrapolated with a population growth rate of 2.8% (13), whereas the polio campaign denominator uses the number of children reached during the previous polio campaign.

**Table 2.** Definition and data of the Madagascar indicators

LQAS indicator	Administrative data indicator
Proportion of children 6–11 mo of age in the probability sample who received a vitamin A supplement during the last campaign (maternal recall, after being shown a vitamin A capsule)	Numerator: number of children 6–11 mo who received a vitamin A supplement during the most recent campaign
	Denominator: number of children 6–11 mo
Proportion of children 12–59 mo of age in the probability sample who received a vitamin A supplement during the last campaign (maternal recall, after being shown a vitamin A capsule)	Numerator: number of children 12–59 mo who received a vitamin A supplement during the most recent campaign
	Denominator: number of children 12–59 mo
Proportion of children 6–11 mo who received polio vaccine during the last campaign (maternal recall)	Numerator: number of children 6–11 mo who received polio vaccine during the most recent campaign
	Denominator: number of children 6–11 mo who received polio vaccine during the previous campaign
Proportion of children 12–59 mo who received polio vaccine during the last campaign (maternal recall)	Numerator: number of children 12–59 mo who received polio vaccine during the last campaign
	Denominator: number of children 12–59 mo who received polio vaccine during the previous campaign

Because the administrative indicators for CHDs in both locations are estimators, they can be larger than 100%. Because they are approximations, assessing their SEs will give a measure of their accuracy.

### The Probability Surveys.

We used stratified random samples assessed with lot quality assurance sampling (LQAS) methodology to measure CHD coverage in each country. The strata were weighted by their population sizes and are known as supervision areas (SAs). They correspond to the CHD teams distributing services. Each commune or district typically has at least five SAs. The first stage sample uses probability proportional to size to randomly sample villages [typically, *n* = 19 (14)]. In each random location a second stage sample identifies an index household using segmentation sampling (15, 16) from locally constructed hand-drawn maps. A random number table is used to select an index house in the segment. To guard against any household having zero probability of selection due to being excluded from the map, the next closest house is selected for interview. Individuals in the two target groups (children 6–11 mo and 12–59 mo) are listed with one selected randomly using a random number table. The remaining target group is selected in the next closest house using the same protocol (17). The resultant sample of individuals is random and thus provides its own SE estimator. One member of each target group only is selected in a sampled village (*SI Appendix* S1, Table S1, for steps used for the sampling).

LQAS surveys use structured questionnaires to measure multiple health indicators. Each indicator is subsequently classified as having reached (or not) a predefined target at the SA level using a statistically determined decision cutoff. This cutoff is associated with the coverage target and acceptable misclassification errors (14).

The higher-level district/commune management unit responsible for local SAs is the catchment area (CA). A CA-level estimator of indicator coverage is a weighted average of the SA estimators, weighted by SA population size. All CA data can also be aggregated to measure coverage in the entire program.

The Benin LQAS health survey was conducted to estimate post-NID coverage (18). Data collection took place during November 16–20, 2015, using mobile devices programmed with Open Data Kit software. Data were collected in CAs in 19 priority communes, each divided into five SAs except for one commune (Ouinhi) which had four SAs. The total random sample size is 1,786 respondents per target group.

The Madagascar LQAS health survey was implemented in three district CAs (Andramasina, Vatomandry, and Miandrivazo) (12). Each district was randomly selected from one of three groupings: low, adequate, and high VAS coverage according to the administrative data. The CHD took place during October 26–30, 2015, with the survey happening during December 8–11, 2015. The districts comprise 12, 15, and 19 SAs. Two SAs in Miandrivazo could not be surveyed, because they were impassable due to heavy rainfall. The total random sample size for each target group was 836.

### Annealing Technique Design.

The LQAS data were annealed to administrative data for four indicators in each country (each response is based on maternal recall within 6 wk of the CHD): the proportion of children in two age groups (6–11 mo and 12–59 mo old) and two indicator groups (those receiving VAS and polio vaccination during the last campaign). We calculate the coverage of each indicator at the CA level (i.e., a commune in Benin and a district in Madagascar).

The combined or hybrid estimator is a linear combination of the two individual coverages, constructed from the following formula:

$$p_{COMBINED\_k}=w_{k}*p_{ADMIN\_k}+\left(1-w_{k}\right)*p_{LQAS\_k},$$

where $p_{COMBINED\_k}$ is the combined coverage estimator in the CA indexed by k, $p_{ADMIN\_k}=Numerator_{ADMIN}/Denominator_{ADMIN}$ is the coverage estimator of the administrative data, $p_{LQAS\_k}$ is the LQAS survey coverage estimator, and $w_{k}$ is a weighting factor between 0 and 1.

The weighting factor $w_{k}$ is also used to calculate the SE of the combined estimator:

$$\text{SE}\left(p_{COMBINED\_k}\right)=\sqrt{w_{k}^{2}*\sigma_{ADMIN\_k}^{2}+{\left(1-w_{k}\right)}^{2}*\sigma_{LQAS\_k}^{2}},$$

where $\sigma_{ADMIN\_k}$ is the SE of $p_{ADMIN\_k}$ and $\sigma_{LQAS\_k}$ is the SE of $p_{LQAS\_k}$.

For this formula to be valid, the two estimators must be uncorrelated. For VAS and polio vaccination campaigns this requirement is satisfied because neither the administrative nor LQAS data utilize information contained in the other; they were two independent measurement events. We use $\text{SE}\left(p_{COMBINED\_k}\right)$ to construct a 95% confidence interval for $p_{COMBINED\_k}$.

### Calculation of $\sigma_{ADMIN\_k}^{2}$ and $w_{k}$.

The squared SEs, $\sigma_{ADMIN\_k}^{2}$ and $\sigma_{LQAS\_k}^{2}$, are variances of each data source estimator and provide the two components of the difference in error between the ADMIN and LQAS estimators. If we average the squared difference, ${\left(p_{ADMIN\_k}-p_{LQAS\_k}\right)}^{2},$ between the two coverage estimators in each CA over all CAs surveyed, we obtain an average of the squared error, called the mean squared error (MSE). Because the administrative data and the LQAS data are collected independently, the MSE is also equal to the sum of the two variances. Hence, we obtain the SE of the administrative coverage from the formula

$$\sigma_{ADMIN\_k}=\sqrt{\text{MSE}-\sigma_{LQAS\_k}^{2}}=\sqrt{\frac{1}{K}\sum_{l=1}^{K}{\left(p_{ADMIN\_l}-p_{LQAS\_l}\right)}^{2}-\sigma_{LQAS\_k}^{2}}\text{,}$$

where K is the total number of CAs surveyed (K=19 for Benin, and K=3 for Madagascar) and k=1,…,K.

We calculate the $\sigma_{ADMIN\_k}$ for each CA, whereas we calculate the MSE once per country, and thus, the MSE has the same value for all CAs surveyed in each country, per intervention.

The combined estimator $\left(p_{COMBINED\_k}\right)$ is a weighted average of two estimators. To minimize the variance of this combined estimator, the positive weights given to each component estimator [$w_{k}$ and (1 − $w_{k}$)] are inversely proportional to the respective variances. They need to sum to 1 so as to retain the unbiasedness of the combined estimator. Thus, the weighting factor $w_{k}=\sigma_{LQAS\_k}^{2}/\left(\sigma_{ADMIN\_k}^{2}+\sigma_{LQAS\_k}^{2}\right)=\sigma_{LQAS\_k}^{2}/\text{MSE}$ and is recalculated for each CA.

For ease of notation, we omit the subscript k in the remaining sections.

### Ethics Statement.

The Ethical Committees of UNICEF NY, UNICEF Benin, UNICEF Madagascar, and the Liverpool School of Tropical Medicine Research Ethics Committee approved the protocol, study instruments, and consent procedures for the LQAS surveys. All interviewees were read an approved informed consent statement indicating the purpose of the survey, the expected amount of time the interview would take, and that participation was voluntary; they were then invited to ask questions about the survey before giving their consent.

## Results

### Benin.

Table 3 shows the MSE for each intervention coverage. The polio and VAS 12–59 mo MSE values are similar, whereas VAS 6–11 is 6–9 times larger. This MSE is much larger because the average LQAS estimate is just over 80% coverage, whereas 18 out of 19 administrative coverage estimates are >100% (*SI Appendix* S2, Table S1). For the other three interventions, the average LQAS estimate ranges from 88.4 to 90.7% coverage, whereas the average administrative estimate ranges from 98.3 to 100.3%, and 29 of the 57 commune administrative coverages are >100% (*SI Appendix* S2, Tables S2–S4).

**Table 3.** Mean squared error (MSE) for the four indicators in 19 communes in Benin and three districts in Madagascar

Indicator	MSE	Number of CAs with $p_{ADMIN}$ > 1	Average value of $p_{ADMIN}$ overall CAs*	Average value of $p_{LQAS}$ overall CAs*
Benin				
VAS 6–11 mo	0.2049	18	1.204	0.804
VAS 12–59 mo	0.0267	12	1.003	0.884
Polio 6–11 mo	0.0344	6	0.983	0.887
Polio 12–59 mo	0.0228	13	1.027	0.907
Madagascar				
VAS 6–11 mo	0.0048	0	0.949	0.938
VAS 12–59 mo	0.0112	1	0.995	0.969
Polio 6–11 mo	0.0045	1	0.977	0.955
Polio 12–59 mo	0.0059	1	0.947	0.953

* Nineteen communes in Benin or three districts in Madagascar.

**Fig. 1.**
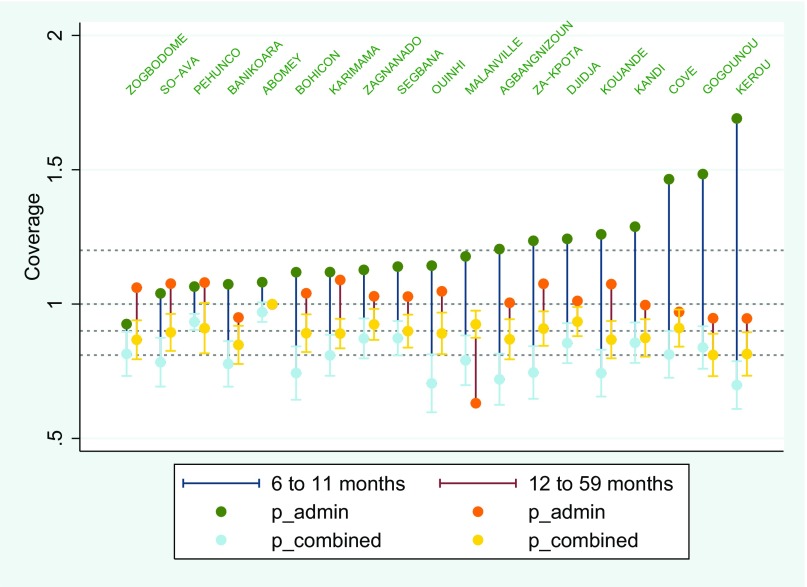
Benin VAS coverage for children aged 6–11 mo and 12–59 mo. For each commune, there are two vertical lines. The left one (dark blue) refers to the 6–11 mo survey, and the right one (dark red) refers to the 12–59 mo survey. For the dark blue line, the dark green balls refer to the administrative estimate (the only ones above 1), and the light blue balls refer to the combined estimator and are almost indistinguishable from the LQAS estimates. Similarly, the dark red bars refer to the 12–59 mo surveys with the orange balls being the administrative estimates and the yellow balls being the combined (and LQAS) estimates. Each combined estimate is displayed with a 95% confidence interval (*SI Appendix* S2, Tables S1 and S2).

**Fig. 2.**
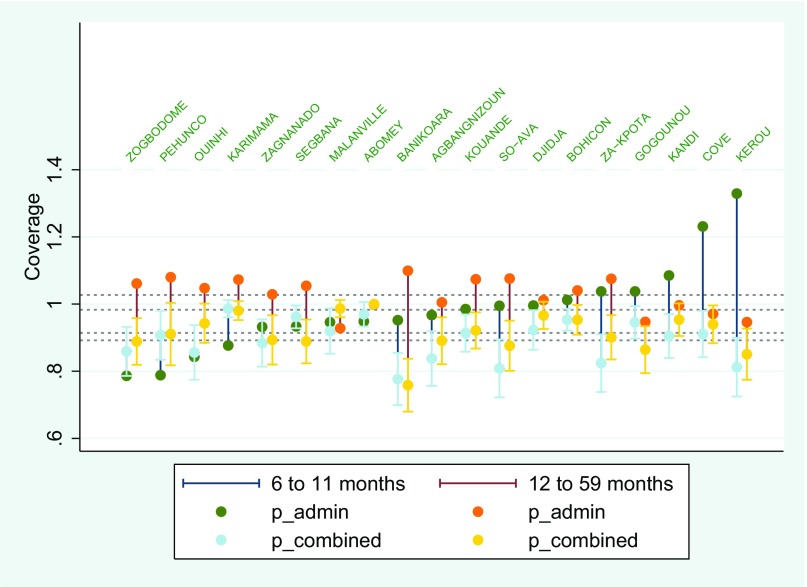
Benin polio vaccination coverage for children aged 6–11 mo and 12–59 mo. For each commune, there are two vertical lines. The left one (dark blue) refers to the 6–11 mo survey, and the right one (dark red) refers to the 12–59 mo survey. For the dark blue line, the dark green balls refer to the administrative estimate (the only ones above 1), and the light blue balls refer to the combined estimator and are almost indistinguishable from the LQAS estimates. Similarly, the dark red bars refer to the 12–59 mo surveys with the orange balls being the administrative estimates and the yellow balls being the combined (and LQAS) estimates. Each combined estimate is displayed with a 95% confidence interval (*SI Appendix* S2, Tables S3 and S4).

The Benin VAS and polio vaccination coverages are given in Figs. 1 and 2, ordered by the administrative estimates. In both sets of surveys, the administrative estimates show a much larger community-to-community variability than the LQAS estimates. Across the two interventions for both age ranges, only 6 of the 19 administrative estimates fall within each respective combined confidence interval.

For VAS 6–11 and 12–59 mo the weighting factor *w* ranges from 0.000 to 0.095, indicating a large discrepancy between the administrative and probability sample estimates. Confidence intervals based on the administrative data are 3.3–25 times wider than those calculated on the basis of the combined estimates. The combined estimates differ from the LQAS coverage estimate by no more than 1%. This results in confidence intervals based on the combined estimates that are slightly tighter than those based on the LQAS estimates alone. For polio 6–11 and 12–59 mo the weighting factor *w* ranges from 0.000 to 0.111. Confidence intervals based on the administrative data are 3–14.1 times wider than those calculated based on the combined estimates. The combined estimates differ from the LQAS coverage estimate by no more than 3% (*SI Appendix* S2, Tables S1–S4). For polio coverage the confidence interval of the hybrid estimate is tighter than the LQAS sample alone.

The ranking of the Benin VAS 6–11 administrative and LQAS coverages is different (Fig. 1). The ratio between the average five highest administrative coverages and the average five lowest administrative coverages is 1.35 and 0.93 for the administrative and LQAS data, respectively. This means that the two sources of data rank the communes very differently (e.g., Kérou and Kouandé) but also hides that most of the administrative coverages are >100%. Capping the 18 VAS 6–11 administrative coverages >100% to 100% would imply the distribution campaign has been extremely successful in accessing the targeted population and obfuscate the actual error. The probability survey data reveal gaps in coverage. Capping all but 1 of the 19 communes shows this readily; accessible routine data mask gaps in service delivery, providing little useful information for future campaigns and current management.

### Madagascar.

In Madagascar the MSE value for VAS 12–59 is 1.9–2.5 times larger than the other three intervention coverages. This occurs because VAS 12–59 includes the largest administrative coverage (112% in Miandrivazo) across the three districts. Across the two interventions for both age groups, LQAS average coverage estimates range from 93.8 to 96.9%, whereas the average administrative coverage average estimates range from 94.7 to 99.5%; at most, one district out of three has an administrative coverage >100% (*SI Appendix* S2, Table S5).

For VAS 6–11 and 12–59 mo, the weighting factor *w* ranges from 0.005 to 0.071. The combined estimates differ from the LQAS coverage estimate by no more than 1%. The confidence intervals are 3.7–13.7 times wider, when based on the administrative data as opposed to the combined estimates. The results from the combined data are very similar to the LQAS results.

For polio 6–11 and 12–59 mo the weighting factor *w* ranges from 0.017 to 0.055 indicating large differences in the variability of the administrative and probability sample estimates. The combined estimates differ from the LQAS coverage estimates by no more than 1%. As for VAS, the confidence intervals are much wider (4.2–7.7 times) when using the administrative data as opposed to the LQAS data. The results from the combined data are very similar to the LQAS results but with slightly less error.

## Discussion

With health systems and major donor programs for health interventions worldwide under severe resourcing constraints, evidence-based data-driven interventions are increasingly required (1, 19). The DHIS2 system now used in much of sub-Saharan Africa is a major step forward in making national data more accessible, but its use as the sole source of information to provide administrative estimates of intervention coverages is problematic. This and other HIS systems, which generally do not produce SEs, are touted as having increasing importance for improving the coverage and quality of health services (20, 21). Their limitations include incomplete data due to health facilities not sending reports regularly; incomplete monthly reporting of all data fields; inaccurate transcription of facility records (22); and for intervention coverage estimates, where both a numerator and a denominator are required, the difficulty with having a good estimator of the denominator. These problems occur in developed and low-resource countries (7), leading some policy-makers to call for a restructuring of information systems (23). Although evolution of these systems will undoubtedly occur, a mechanism is required now to improve our estimates of risk associated with using them for large-scale intervention coverage estimation. This paper provides a mechanism to satisfy this need while producing a more accurate estimate of coverage. Our hybrid estimator can accelerate the evolution of HIS by identifying a weakness and its magnitude.

Administrative estimators have been mistrusted by national and international decision-makers due to their apparent inaccuracy (6); we reach a similar conclusion when contrasting CHD data to probability survey data. Estimates drawn from the DHIS2 also exhibit large variability from one locale to another, even for proximal locations. Multiple ad hoc remedies have been proposed to overcome these deficiencies (24, 25), but these do not provide a consistent approach that can be used by ministries of health to improve the coverage information on which decisions are made. The hybrid estimator proposed here is a statistically principled solution that has the benefit of estimating its own accuracy and the error in administrative CHD data, which was previously unknown. This approach is also applicable to facility-based DHIS2 data, which is another class of administrative data collected using facility registries. The hybrid estimator depends on having, as a minimum, a relatively small set of survey data available. We used LQAS data to demonstrate this point. Timely, larger data sources, such as those from the Demographic and Health Surveys, or the Multi Indicator Cluster Surveys could also be used.

Our approach differs from WHO’s attempt to achieve this with computation logic, where the estimates still rely on professional judgments and do not produce error terms or a measure of risk (24). The Institute of Health Metrics and Evaluation combines data using more complex quantitative analysis rather than human judgments. It produces an estimate of the number of additional children covered or overestimated (25). However, like computational logic, it does not measure error. The two methods are comparable deterministic methods (24).

Our combined estimator calculates error for all data sources and combinations. It is linear and uses a weighting factor w inversely proportional to the estimated mean squared error of each separate estimator. The weight w appraises the microgeographical or SA-level variability measured by the LQAS surveys; if the probability survey is smaller than the macrogeographical or CA-level variability of the disagreement between the LQAS and administrative coverage estimators, then the weighting factor *w* is closer to 0. If it is the reverse, then the weighting factor *w* is closer to 1. In principle, they should agree because both should represent the population. If such is the case, then the weighting factor *w* is close to 0.50.

For polio vaccination and VAS interventions for two age groups in Benin and Madagascar the observed variation between the administrative coverage estimate was much larger than the variation between SAs comprising the CAs, with the result that the weighting factor *w* was closer to 0. The DHIS2 data variability within a country is suspect when the survey data does not exhibit the same pattern. As a result, the combined estimate has a value closer to the probability sample estimation. The benefit derived from this combining of the two estimators is a more accurate estimation of the intervention than either the administrative or the survey data alone. This improved accuracy, when tracking health systems, will improve even further as the administrative systems improve.

This study demonstrates that even small probability samples, when combined with administrative data, can produce a more accurate estimate of coverage and calculate a measure of the error of the HIS. It provides a principled recipe for combining probability surveys with administrative datasets, which can improve the utility of DHIS2 as it is rolled out across low resource countries. We recommend that this hybrid prevalence estimator be incorporated into the DHIS2 to appraise data quality and guide decision-makers in their principled use of HIS data.

## Supplementary Material

Supplementary File

## References

[1] Chan M (2010). Meeting the demand for results and accountability: A call for action on health data from eight global health agencies. PLoS Med.

[2] Organization WH (2007). Sixtieth World Health Assembly: Resolution and Decisions, Annexes.

[3] Frieden TR (2015). Shattuck Lecture: The future of public health. N Engl J Med.

[4] Maina I (2017). Using health-facility data to assess subnational coverage of maternal and child health indicators, Kenya. Bull World Health Organ.

[5] Admon R, Milad MR, Hendler T (2013). A causal model of post-traumatic stress disorder: Disentangling predisposed from acquired neural abnormalities. Trends Cogn Sci.

[6] Aqil A, Lippeveld T, Hozumi D (2009). PRISM framework: A paradigm shift for designing, strengthening and evaluating routine health information systems. Health Policy Plan.

[7] Woodworth GF, Baird CJ, Garces-Ambrossi G, Tonascia J, Tamargo RJ (2009). Inaccuracy of the administrative database: Comparative analysis of two databases for the diagnosis and treatment of intracranial aneurysms. Neurosurgery.

[8] Pagano M, Gauvreau K (2000). Principles of Biostatistics.

[9] Hedt BL, Pagano M (2011). Health indicators: Eliminating bias from convenience sampling estimators. Stat Med.

[10] Anoke SC, Mwai P, Jeffery C, Valadez JJ, Pagano M (2015). Comparing two survey methods of measuring health-related indicators: Lot quality assurance sampling and demographic health surveys. Trop Med Int Health.

[11] Valadez JJ, Jeffery C, Brant T, Vollmer N (2017).

[12] Cisse M, Vargas W, Jeffery C (2016). Évaluation de la validité des données administratives de la Semaine de la Santé de la Mère et de l’enfant (SSME) d’Octobre 2015 dans trois (3) Districts de Madagascar.

[13] United Nations Development Programme (2017). http://www.mg.undp.org/content/madagascar/fr/home/countryinfo.html.

[14] Robertson SE, Valadez JJ (2006). Global review of health care surveys using lot quality assurance sampling (LQAS), 1984-2004. Soc Sci Med.

[15] Turner AG, Magnani RJ, Shuaib M (1996). A not quite as quick but much cleaner alternative to the Expanded Programme on Immunization (EPI) cluster survey design. Int J Epidemiol.

[16] Davis RH, Valadez JJ (2014). Improving the collection of knowledge, attitude and practice data with community surveys: A comparison of two second-stage sampling methods. Health Policy Plan.

[17] Valadez JJ, Weiss W, Leburg C, Davis R (2007). Assessing Community Health Programs: A Trainer’s Guide. Using LQAS for Baseline Surveys and Regular Monitoring.

[18] Gbangbade S, Vargas W, Jeffery C (2016).

[19] Boerma T (2014). Monitoring progress towards universal health coverage at country and global levels. PLoS Med.

[20] Campbell B (1997). Health Management Information Systems in Lower Income Countries: An Analysis of System Design, Implementation and Utilization in Ghana and Nepal.

[21] Lippeveld T, Sauerborn R, Bodart C (2000). Design and Implementation of Health Information Systems.

[22] Chaulagai CN (2005). Design and implementation of a health management information system in Malawi: Issues, innovations and results. Health Policy Plan.

[23] Lippeveld T, Sauerborn R, Sapirie S (1997). Health information systemsMaking them work. World Health Forum.

[24] Burton A, Kowalski R, Gacic-Dobo M, Karimov R, Brown D (2012). A formal representation of the WHO and UNICEF estimates of national immunization coverage: A computational logic approach. PLoS One.

[25] Lim SS, Stein DB, Charrow A, Murray CJ (2008). Tracking progress towards universal childhood immunisation and the impact of global initiatives: A systematic analysis of three-dose diphtheria, tetanus, and pertussis immunisation coverage. Lancet.

