# Supplementary material for: Hybrid prevalence estimation: Method to improve intervention coverage estimations
Source: Proc Natl Acad Sci U S A. 2018 Dec 5;115(51):13063–8. doi: 10.1073/pnas.1810287115 (PMC6304954; doi:10.1073/pnas.1810287115)
Supplement: Supplementary File [file pnas.1810287115.sapp.pdf]

Supplementary Information 1

Table S1: Sequential steps in the protocol used to sample the LQAS data in Benin and Madagascar

1. Catchment areas (SA) are defined as Districts or Communes.
2. Strata within each CA are identified, which are administrative units responsible for service delivery. Strata are referred to as supervision areas (SA).
3. A sampling frame is constructed for each SA consisting of villages and their population sizes.
4. The stage sample is a probability proportional to size sample of 19 random locations, which are often 19 different villages, in each SA.
5. In each random location, a second stage sample identifies an index household. Using a hand drawn map, the village is subdivided into segments of approximately the same size and numbered. One segment is selected randomly using a random number table.
6. If the segment has more than approximately 20 households, it is also divided into segments as in step 5, and one is selected randomly. This procedure continues until one segment is selected with approximately 20 or fewer households.
7. The households in the selected segment are enumerated and one is randomly selected using a random number table.
8. The first household is used as an index household. To guard against any household having zero probability of selection due to being excluded from the map, the next closest house is selected as a candidate for interview.
9. Individuals in the two target groups (children 6-11 months and 12-59 months) are listed with one child selected randomly using a random number table.
10. The remaining target group is selected in the next closest house using the same protocol. The children in the age group are listed and one is selected randomly with a random number table. If the next closest house does not have the target group, the interviewer proceeds to the subsequent next closest door until a target group member is identified.
11. All 19 locations are sample using the same protocol in all strata.

www.pnas.org/cgi/doi/10.1073/pnas.1810287115

Supplementary Information 2

Results

A. *Benin*

For VAS 6-11 months, the weighting factor *w* ranges from 0.001 to 0.015 (Table S1). The combined estimate differs from the LQAS coverage estimate by no more than 1%. Confidence intervals based on the administrative data are 8.2 to almost 25 times wider than those calculated on the basis of the combined estimates (Table S1, last column).
For VAS 12-59 months, the weighting factor *w* ranges from 0.000 to 0.095 (Table S2). The combined estimate differs from the LQAS coverage estimate by no more than 1%. Confidence intervals based on the administrative data are 3.3 to almost 6.3 times wider than those calculated on the basis of the combined estimates (Table S2, last column).
For polio 6-11 months, the weighting factor *w* ranges from 0.005 to 0.062 (Table S3). The combined estimate differs from the LQAS coverage estimate by no more than 3%. Confidence intervals based on the administrative data are 4.1 to almost 14.1 times wider than those calculated on the basis of the combined estimates (Table S3, last column).
For polio 12-59 months, the weighting factor *w* ranges from 0.000 to 0.111 (Table S4). The administrative SE is between 2.8 to 11.4 times larger than the LQAS SE. The combined estimate differs from the LQAS coverage estimate by no more than 3%. Confidence intervals based on the administrative data are 3 to almost 11.5 times wider than those calculated on the basis of the combined estimates (Table S4, last column).

Table S1: Benin coverage proportion of children 6-11 months who received a vitamin A supplement during the last campaign

Commune	p_{ADMIN}	σ_{ADMIN}	p_{LQAS}	σ_{LQAS}	w	$p_{COMBINED}$	95% CI †	Relative width between CIs ††
ABOMEY	1.08	0.4523	0.97	0.0186	0.002	0.97	(0.93,1)	24.32
AGBANGNIZOUN	1.20	0.4500	0.71	0.0489	0.012	0.72	(0.62,0.82)	9.25
BANIKOARA	1.07	0.4506	0.77	0.0434	0.009	0.78	(0.69,0.86)	10.42
BOHICON	1.12	0.4498	0.74	0.0511	0.013	0.74	(0.64,0.84)	8.87
COVE	1.46	0.4505	0.81	0.0445	0.010	0.81	(0.73,0.9)	10.16
DJIDJA	1.24	0.4510	0.85	0.0382	0.007	0.85	(0.78,0.93)	11.86
GOGOUNOU	1.48	0.4508	0.83	0.0406	0.008	0.84	(0.76,0.92)	11.15
KANDI	1.29	0.4510	0.85	0.0386	0.007	0.86	(0.78,0.93)	11.73
KARIMAMA	1.12	0.4510	0.81	0.0390	0.007	0.81	(0.73,0.89)	11.59
KEROU	1.69	0.4504	0.69	0.0456	0.010	0.70	(0.61,0.79)	9.93
KOUANDE	1.26	0.4504	0.74	0.0446	0.010	0.74	(0.66,0.83)	10.15
MALANVILLE	1.18	0.4502	0.79	0.0474	0.011	0.79	(0.7,0.88)	9.54
OUINHI	1.14	0.4492	0.70	0.0554	0.015	0.70	(0.6,0.81)	8.17
PEHUNCO	1.07	0.4524	0.93	0.0153	0.001	0.93	(0.9,0.96)	29.66
SEGBANA	1.14	0.4515	0.87	0.0327	0.005	0.87	(0.81,0.94)	13.83
SO-AVA	1.04	0.4502	0.78	0.0466	0.011	0.78	(0.69,0.87)	9.72
ZA-KPOTA	1.24	0.4498	0.74	0.0503	0.012	0.75	(0.65,0.84)	9.00
ZAGNANADO	1.13	0.4510	0.87	0.0381	0.007	0.87	(0.8,0.95)	11.89
ZOGBODOME	0.93	0.4507	0.81	0.0420	0.009	0.81	(0.73,0.9)	10.77

† The confidence interval (CI) is calculated as $p_{COMBINED} \pm 1.96 * SE(p_{COMBINED})$. (See Methods).
†† The relative width between the confidence intervals of the administrative and combined estimates is equal to $\sigma_{ADMIN} / SE(p_{COMBINED})$.

Table S2: Benin coverage proportion of children 12-59 months who received a vitamin A supplement during the last campaign

Commune	p_{ADMIN}	σ_{ADMIN}	p_{LQAS}	σ_{LQAS}	w	$p_{COMBINED}$	95% CI †	Relative width between CIs ††
ABOMEY	1.00	0.1633	1.00	0.000	0.000	1.00	(1,1)	
AGBANGNIZOUN	1.00	0.1585	0.86	0.039	0.058	0.87	(0.79,0.94)	4.17
BANIKOARA	0.95	0.1590	0.84	0.037	0.052	0.85	(0.78,0.92)	4.38
BOHICON	1.04	0.1591	0.88	0.037	0.050	0.89	(0.82,0.96)	4.45
COVE	0.97	0.1593	0.91	0.036	0.049	0.91	(0.84,0.98)	4.53
DJIDJA	1.01	0.1609	0.93	0.028	0.030	0.93	(0.88,0.99)	5.81
GOGOUNOU	0.95	0.1579	0.80	0.042	0.065	0.81	(0.73,0.89)	3.92
KANDI	1.00	0.1592	0.87	0.036	0.050	0.87	(0.8,0.94)	4.49
KARIMAMA	1.09	0.1608	0.88	0.028	0.030	0.89	(0.84,0.94)	5.74
KEROU	0.95	0.1576	0.80	0.043	0.068	0.81	(0.73,0.89)	3.83
KOUANDE	1.07	0.1592	0.86	0.036	0.050	0.87	(0.8,0.94)	4.49
MALANVILLE	0.63	0.1612	0.93	0.026	0.025	0.92	(0.87,0.98)	6.26
OUINHI	1.05	0.1582	0.88	0.041	0.062	0.89	(0.81,0.97)	4.01
PEHUNCO	1.08	0.1554	0.89	0.050	0.095	0.91	(0.82,1)	3.25
SEGBANA	1.03	0.1602	0.89	0.032	0.038	0.90	(0.84,0.96)	5.11
SO-AVA	1.08	0.1593	0.89	0.036	0.049	0.89	(0.83,0.96)	4.54
ZA-KPOTA	1.08	0.1599	0.90	0.033	0.042	0.91	(0.84,0.97)	4.89
ZAGNANADO	1.03	0.1605	0.92	0.030	0.034	0.92	(0.87,0.98)	5.44
ZOGBODOME	1.06	0.1588	0.86	0.038	0.054	0.87	(0.79,0.94)	4.30

† The confidence interval (CI) is calculated as $p_{COMBINED} \pm 1.96 * SE(p_{COMBINED})$. (See Methods).

†† The relative width between the confidence intervals of the administrative and combined estimates is equal to $\sigma_{ADMIN} / SE(p_{COMBINED})$.

Table S3: Benin coverage proportion children 6–11 months who received polio vaccine during the last campaign

**Commune**	p_{ADMIN}	σ_{ADMIN}	p_{LQAS}	σ_{LQAS}	w	$p_{COMBINED}$	95% CI †	Relative width between CIs ††
ABOMEY	0.95	0.1845	0.97	0.0186	0.010	0.97	(0.93,1)	9.96
AGBANGNIZOUN	0.97	0.1805	0.83	0.0426	0.053	0.84	(0.76,0.92)	4.36
BANIKOARA	0.95	0.1809	0.77	0.0407	0.048	0.78	(0.7,0.85)	4.56
BOHICON	1.01	0.1847	0.95	0.0167	0.008	0.95	(0.92,0.99)	11.12
COVE	1.23	0.1819	0.90	0.0359	0.038	0.91	(0.84,0.98)	5.16
DJIDJA	1.00	0.1829	0.92	0.0305	0.027	0.92	(0.86,0.98)	6.09
GOGOUNOU	1.04	0.1838	0.94	0.0249	0.018	0.95	(0.9,0.99)	7.43
KANDI	1.08	0.1823	0.90	0.0338	0.033	0.90	(0.84,0.97)	5.49
KARIMAMA	0.88	0.1850	0.99	0.0132	0.005	0.99	(0.96,1)	14.05
KEROU	1.33	0.1796	0.78	0.0460	0.062	0.81	(0.72,0.9)	4.03
KOUANDE	0.98	0.1832	0.91	0.0286	0.024	0.91	(0.86,0.97)	6.48
MALANVILLE	0.95	0.1821	0.92	0.0351	0.036	0.92	(0.85,0.99)	5.28
OUINHI	0.84	0.1805	0.86	0.0427	0.053	0.86	(0.77,0.94)	4.35
PEHUNCO	0.79	0.1815	0.91	0.0381	0.042	0.91	(0.83,0.98)	4.87
SEGBANA	0.93	0.1846	0.96	0.0173	0.009	0.96	(0.93,1)	10.70
SO-AVA	0.99	0.1798	0.80	0.0455	0.060	0.81	(0.72,0.89)	4.07
ZA-KPOTA	1.04	0.1799	0.81	0.0451	0.059	0.82	(0.74,0.91)	4.11
ZAGNANADO	0.93	0.1818	0.88	0.0366	0.039	0.88	(0.81,0.95)	5.07
ZOGBODOME	0.79	0.1815	0.86	0.0380	0.042	0.86	(0.79,0.93)	4.88

† The confidence interval (CI) is calculated as $p_{COMBINED} \pm 1.96 * SE(p_{COMBINED})$. (See Methods).
†† The relative width between the confidence intervals of the administrative and combined estimates is equal to $\sigma_{ADMIN} / SE(p_{COMBINED})$.

Table S4: Benin coverage proportion of children 12–59 months who received polio vaccine during the last campaign

**Commune**	p_{ADMIN}	σ_{ADMIN}	p_{LQAS}	σ_{LQAS}	w	$p_{COMBINED}$	95% CI †	Relative width between CIs ††
ABOMEY	1.00	0.1510	1.00	0.0000	0.000	1.00	(1,1)	
AGBANGNIZOUN	1.00	0.1464	0.88	0.0368	0.060	0.89	(0.82,0.96)	4.10
BANIKOARA	1.10	0.1451	0.73	0.0418	0.077	0.76	(0.68,0.84)	3.61
BOHICON	1.04	0.1492	0.95	0.0229	0.023	0.95	(0.91,1)	6.59
COVE	0.97	0.1481	0.94	0.0292	0.037	0.94	(0.88,1)	5.17
DJIDJA	1.01	0.1495	0.97	0.0210	0.019	0.97	(0.93,1)	7.20
GOGOUNOU	0.95	0.1464	0.86	0.0367	0.059	0.86	(0.79,0.93)	4.11
KANDI	1.00	0.1489	0.95	0.0251	0.028	0.95	(0.9,1)	6.02
KARIMAMA	1.07	0.1503	0.98	0.0144	0.009	0.98	(0.95,1)	10.46
KEROU	0.95	0.1455	0.84	0.0403	0.071	0.85	(0.77,0.93)	3.75
KOUANDE	1.07	0.1484	0.92	0.0278	0.034	0.92	(0.87,0.97)	5.42
MALANVILLE	0.93	0.1504	0.99	0.0132	0.008	0.99	(0.96,1)	11.47
OUINHI	1.05	0.1478	0.94	0.0305	0.041	0.94	(0.88,1)	4.95
PEHUNCO	1.08	0.1423	0.89	0.0503	0.111	0.91	(0.82,1)	3.00
SEGBANA	1.05	0.1470	0.88	0.0342	0.051	0.89	(0.82,0.95)	4.42
SO-AVA	1.08	0.1457	0.86	0.0395	0.068	0.88	(0.8,0.95)	3.83
ZA-KPOTA	1.08	0.1469	0.89	0.0346	0.053	0.90	(0.83,0.97)	4.36
ZAGNANADO	1.03	0.1459	0.88	0.0388	0.066	0.89	(0.82,0.97)	3.89
ZOGBODOME	1.06	0.1465	0.88	0.0365	0.058	0.89	(0.82,0.96)	4.14

† The confidence interval (CI) is calculated as $p_{COMBINED} \pm 1.96 * SE(p_{COMBINED})$. (See Methods).
†† The relative width between the confidence intervals of the administrative and combined estimates is equal to $\sigma_{ADMIN} / SE(p_{COMBINED})$.

B. Madagascar

Table S5: Madagascar coverages and standard errors for all four indicators for administrative and LQAS estimate, coverage of combined estimates with 95% CI, relative width between CIs.

	District	p_{ADMIN}	σ_{ADMIN}	p_{LQAS}	σ_{LQAS}	w	$p_{COMBINED}$	95% CI†	Relative width between CIs ††
VAS 6-11 mo	ANDRAMASINA	0.88	0.0675	0.96	0.0145	0.044	0.96	(0.93,0.99)	4.76
	VATOMANDRY	1.00	0.0676	0.93	0.0138	0.040	0.93	(0.91,0.96)	5.00
	MIANDRIVAZO	0.97	0.0665	0.92	0.0184	0.071	0.92	(0.89,0.96)	3.74
VAS 12-59 mo	ANDRAMASINA	0.92	0.1049	0.98	0.0129	0.015	0.97	(0.95,1)	8.21
	VATOMANDRY	0.95	0.1054	0.98	0.0077	0.005	0.98	(0.96,0.99)	13.69
	MIANDRIVAZO	1.12	0.1050	0.95	0.0122	0.013	0.95	(0.93,0.98)	8.70
Polio 6-11 mo	ANDRAMASINA	0.98	0.0643	0.98	0.0098	0.023	0.98	(0.96,1)	6.66
	VATOMANDRY	0.92	0.0652	0.96	0.0110	0.028	0.96	(0.94,0.98)	6.02
	MIANDRIVAZO	1.03	0.0738	0.92	0.0178	0.055	0.93	(0.89,0.96)	4.27
Polio 12-59 mo	ANDRAMASINA	0.91	0.0701	0.95	0.0174	0.058	0.94	(0.91,0.98)	4.15
	VATOMANDRY	0.90	0.0745	0.97	0.0098	0.017	0.97	(0.95,0.99)	7.69
	MIANDRIVAZO	1.04	0.0879	0.94	0.0133	0.022	0.94	(0.92,0.97)	6.68

† The confidence interval (CI) is calculated as $p_{COMBINED} \pm 1.96 * SE(p_{COMBINED})$. (See Methods).
†† The relative width between the confidence intervals of the administrative and combined estimates is equal to $\sigma_{ADMIN} / SE(p_{COMBINED})$.

For VAS 6-11 months, the weighting factor w ranges from 0.04 to 0.071 (Table S5). The combined estimate differs from the LQAS coverage estimate by no more than 1%. Confidence intervals based on the administrative data are between 3.7 to almost five times wider than those based on the combined estimate.
For VAS 12-59 months, the weighting factor *w* ranges from 0.005 to 0.015. The combined estimate differs from the LQAS coverage estimate by no more than 1%. Confidence intervals based on the administrative data are between 8.2 to almost 13.7 times wider than those based on the combined estimate.
For polio 6-11 months, the weighting factor *w* ranges from 0.023 to 0.055. The combined estimate differs from the LQAS coverage estimate by no more than 1%. Confidence intervals based on the administrative data are between 4.3 to almost 6.7 times wider than those based on the combined estimate.
For polio 12-59 months, the weighting factor w ranges from 0.017 to 0.055. The combined estimate differs from the LQAS coverage estimate by no more than 1%. Confidence intervals based on the administrative data are between 4.2 to almost 7.7 times wider than those based on the combined estimate.
